# Cappable-seq RNA-sequencing data sets for comparative studying of *Salmonella enterica* adaptation to *Acanthamoeba castellanii* intracellular environment, oxidative stress, and starvation

**DOI:** 10.1128/mra.01129-24

**Published:** 2025-05-28

**Authors:** Alexander Balkin, Andrey Plotnikov, Tatiana Konnova, Natalia Gogoleva, Elena Shagimardanova, Yuri Gogolev

**Affiliations:** 1 Kazan Institute of Biochemistry and Biophysics, Kazan Scientific Center of Russian Academy of Sciences, Kazan, Russia; 2 Institute for Cellular and Intracellular Symbiosis, Ural Branch of the Russian Academy of Sciences, Orenburg, Russia; 3 Institute of Fundamental Medicine and Biology, Kazan Federal University 64922, Kazan, Russia; 4 Research Department for Limnology, Mondsee, Universität Innsbruck, Mondsee, Austria; 5 Loginov Moscow Clinical Scientific Center, Moscow, Russia; University of Maryland School of Medicine, Baltimore, Maryland, USA

**Keywords:** Cappable-seq transcriptome, *Salmonella enterica*, *Acanthamoeba castellanii*, oxidative stress, starvation

## Abstract

*Salmonella* is a foodborne pathogen that can survive various stresses and replicate inside protist cells. Here, we present high-resolution Cappable-seq transcriptomic data for *Salmonella enterica* 14028S under starvation and hydrogen peroxide treatment, as well as 8 and 15 h after *Acanthamoeba castellanii* uptake.

## ANNOUNCEMENT

The ability of *Salmonella* to colonize protozoa as a natural reservoir deserves careful study (1). The Cappable-seq method (2) enables the analysis of bacterial transcriptomes within eukaryotic cells (3). Here, we present a Cappable-Seq data set for *Salmonella* surviving in *Acanthamoeba* at 8 and 15 hours post-infection (hpi), free-living bacteria in poor medium, rich medium, and under hydrogen peroxide treatment.

*Salmonella enterica* 14028S cells were grown in 10 mL Luria-Bertani broth (LB) to mid-log phase (OD_600_ = 0.4) and either harvested directly (control), treated with 1 mM hydrogen peroxide for 20 min, or washed three times and cultured for 2 h in Page’s amoeba saline (PAS) medium (4) to induce starvation.

*Acanthamoeba castellanii* Neff (ATCC 30010) cells were grown for 7 days in peptone‐yeast extract‐glucose (PYG) medium (5) at 27°С to 4–5 × 10^5^ cells/mL, collected by centrifugation at 800 g for 5 min, washed twice, and suspended in 10 mL of PAS. *Salmonella* cells grown in LB to stationary phase (OD_600_ = 2.0) were harvested by centrifugation, washed twice with PAS, and added to the *Acanthamoeba* cultures at a bacteria:ameba cells ratio of 100:1. After 1 hour of incubation, the medium was supplemented with gentamicin to 100 µg × mL^−1^, and after an additional hour, it was replaced with PAS containing 10 µg × mL^−1^ gentamicin. At 8 and 15 hpi, when *Acanthamoeba* began to die, the amebae cells were collected by centrifugation. After careful removal of the supernatant, the cells were suspended in 10 mL of ice-cold 0.2% SDS, 19% ethanol, and 1% acidic phenol and incubated for 30 min. After ameba cell lysis, *Salmonella* cells were collected by centrifugation at 3,000 g, 4°C, for 5 min.

Total RNA was extracted from the bacterial pellets using RNA Extract Reagent (Evrogen, Moscow, Russia), purified using the RNase-Free DNase I kit (Ambion, Austin, TX, USA), and assessed with a Bioanalyzer 2100 (Agilent, Santa Clara, CA, USA). The purified RNA from *Salmonella* (5 µg) or bacterial-amebic samples (10 µg) was capped with 3′ desthiobiotin-GTP using the Vaccinia Capping System (New England Biolabs, Ipswich, MA, USA), purified by Clean and Concentrator™−5 column (Zymo Research, Irvine, CA, USA), and fragmented by heating at 94°C for 5 min in polynucleotide kinase buffer. The 3′-phosphates were removed with T4 polynucleotide kinase. The capped RNA was captured with streptavidin magnetic beads (NEB), eluted with biotin, and purified using AMPure XP beads (Beckman Coulter, Brea, CA, USA).

Libraries were generated using the NEBNext small RNA library prep kit (NEB), assessed using a Bioanalyzer 2100 (Agilent), and quantified by qPCR. Sequencing (75 bp paired-end) was performed on the NextSeq 550 instrument (Illumina, San Diego, CA, USA).

**Fig 1:**
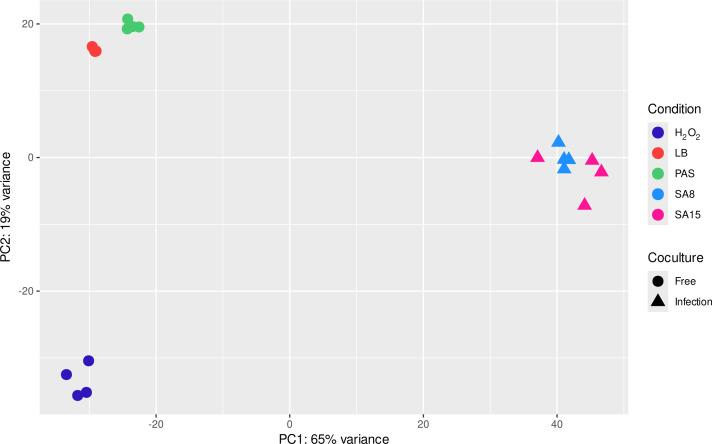
PCA plot of *Salmonella* Cappable-seq data, showing sample–sample distances of *Salmonella* cocultured with *Acanthamoeba* (SA8 and SA15) samples and free-living cultures (LB, PAS, H_2_O_2_).

When processing data, the default program parameters were used unless otherwise stated. Reads were aligned to the *A. castellanii* genome (GCA_000313135) and *S. enterica* 14,028s genome (GCA_000022165.1) (6) using STAR aligner v2.7.11a (7) (https://github.com/alexdobin/STAR). The featureCounts v1.4.6-p5 function of Rsubread package version 2.14.2 (8) (http://subread.sourceforge.net) was used to quantify the number of reads mapped to each gene. A summary of reads is presented in Table 1. Principal component analysis (Fig. 1) was performed using DESeq2 (9).

**TABLE 1:** Summary of sequencing reads^*a*^

Sample	*S. enterica* cultivation conditions	No. of reads	% of reads mapped to *A*. *castellanii* genome	% of reads mapped to *S. enterica* genome	% of reads mapped to rRNA	SRA accession no.	GEO accession no.
LB1	Control	12483741	0.08	98.87	3.92	SRR29681637	GSM8374379
LB2		13027068	0.23	98.14	4.91	SRR29681636	GSM8374380
LB3		12841679	0.13	98.88	5.77	SRR29681635	GSM8374381
LB4		19382924	0.11	98.79	4.99	SRR29681634	GSM8374382
PAS1	Starvation	9341414	0.1	97.83	5.65	SRR29681633	GSM8374383
PAS2		19736873	0.04	99.03	7.65	SRR29681632	GSM8374384
PAS3		19487723	0.04	98.72	6.59	SRR29681631	GSM8374385
PAS4		19970809	0.06	97.98	6.41	SRR29681630	GSM8374386
HP1	Hydrogen peroxide treatment	22431992	0.27	98.94	8.44	SRR29681641	GSM8374375
HP2		26907293	0.15	99.29	7.01	SRR29681640	GSM8374376
HP3		20991379	0.22	98.95	7.73	SRR29681639	GSM8374377
HP4		16397152	0.31	98.59	4.93	SRR29681638	GSM8374378
SA8-1	Cocultivation with *A*. *castellanii* 8 hpi	9101379	7.08	74.91	4.95	SRR29681629	GSM8374387
SA8-2		8056006	7.75	68.7	7.03	SRR29681628	GSM8374388
SA8-3		9117165	4.48	80.27	6.26	SRR29681627	GSM8374389
SA8-4		12520573	5.91	78.85	5.18	SRR29681626	GSM8374390
SA15-1	Cocultivation with *A*. *castellanii* 15 hpi	14017645	1.85	92.99	9.33	SRR29681625	GSM8374391
SA15-2		10747794	1.61	94.31	9.4	SRR29681624	GSM8374392
SA15-3		20477512	2.04	92.28	10.57	SRR29681623	GSM8374393
SA15-6		15063175	4.64	86.02	4.75	SRR29681622	GSM8374394

^*a*^ The numbers of reads after their filtering and mapping to the reference genome are given. For all libraries, the read quality corresponded to the parameter Q > 30 on the Phred scale.

## Data Availability

The Cappable-seq raw reads have been deposited in the NCBI SRA database and are accessible through BioProject accession no. PRJNA1130907. The transcriptomic data have been deposited in the NCBI GEO database under data set accession no. GSE270532. The processed samples and their accession numbers are listed in Table 1. The announced Cappable-seq data provide a resource for mapping new transcription start sites, detecting non-coding RNA activity and dual transcriptome analysis of *S. enterica* in *A. castellanii*.
